# Investigating effects of preoperative inflammatory biomarkers on predicting survival outcomes of intrahepatic cholangiocarcinoma after curative resection

**DOI:** 10.1186/s12957-020-02053-w

**Published:** 2020-10-23

**Authors:** Zeyu Zhang, Yufan Zhou, Kuan Hu, Yun Huang

**Affiliations:** Department of Hepatobiliary Surgery, Xiangya Hospital, Central South University, Changsha, Hunan China

**Keywords:** Intrahepatic cholangiocarcinoma, Inflammatory biomarker, Systemic immune-inflammation index

## Abstract

**Introduction:**

Intrahepatic cholangiocarcinoma (ICC) stands as the second most common malignant tumor in the liver with poor patient prognosis. Increasing evidences have shown that inflammation plays a significant role in tumor progression, angiogenesis, and metastasis. However, the prognosis significance of inflammatory biomarkers on recurrence-free survival (RFS) and overall survival (OS) in ICC patients is poorly recognized.

**Methods:**

ICC patients who underwent curative hepatectomy and diagnosed pathologically were retrospectively analyzed. Inflammatory biomarkers, including neutrophil-to-lymphocyte ratio (NLR), platelet-to-lymphocyte ratio (PLR), lymphocyte-to-monocyte ratio (LMR), and systemic immune-inflammation index (SII), were investigated.

**Results:**

Receiver operating characteristic (ROC) curves showed no significance in NLR, PLR, and LMR in RFS and OS, while significant results were shown on SII in both RFS (*P* = 0.035) and OS (*P* = 0.034) with areas under ROC curve as 0.63 (95%CI 0.52–0.74) and 0.62 (95%CI 0.51–0.72), respectively. Kaplan-Meier curves revealed a statistically significant better survival data in SII-low groups on both RFS (*P* < 0.001) and OS (*P* < 0.001). The univariate and multivariate analyses revealed that higher level of SII was independently associated with both poorer RFS time and OS time. However, no significant result was shown on NLR, PLR, or LMR.

**Conclusion:**

SII is an effective prognostic factor for predicting the prognosis of ICC patient undergone curative hepatectomy, while NLR, PLR, and LMR are not associated with clinical outcomes of these patients.

**Supplementary Information:**

The online version contains supplementary material available at 10.1186/s12957-020-02053-w.

## Introduction

Intrahepatic cholangiocarcinoma (ICC) stands as the second common malignant hepatic neoplasms; however, the incidence of ICC grows worldwide during past decades [1, 2]. Up to now, the best choice of curative treatments is surgical resection, while the treatments for unresectable ICC are very limited [3]. ICC usually grows aggressively without symptom in early stage, resulting in a small proportion of ICC patients who can receive surgery. Furthermore, the prognosis of resectable ICC patient still remains poor and half of them will suffer from recurrence after surgery [4]. Many researches were performed and found various significant risk factors in ICC, helping the management of ICC patients [5, 6].

Meantime, increasing evidences have shown that inflammation and inflammatory biomarkers are significant factors in tumor microenvironment, thus promoting proliferation, angiogenesis, and metastasis by various inflammatory cells and cytokines [7]. In recent years, multiple inflammatory biomarkers were investigated for predicting the prognosis of patients with various cancer, including neutrophil-to-lymphocyte ratio (NLR), platelet-to-lymphocyte ratio (PLR), lymphocyte-to-monocyte ratio (LMR), and systemic immune-inflammation index (SII). Significant results were reported in various types of cancer such as neck soft tissue sarcoma, lung cancer, renal cancer, and hepatocellular carcinoma [8–10].

However, the significance of these inflammatory biomarkers on prognosis of ICC patients underwent curative resection has not been fully understood. Therefore, the present study was performed for investigating the significance of various inflammatory biomarkers, including NLR, PLR, LMR, and SII on patient prognosis in ICC after curative surgery.

## Methods

### Study cohort

ICC patients underwent curative hepatectomy between January 2013 and December 2017 in Xiangya hospital, Central South University, were retrospectively analyzed. Exclusion criteria were as followed: (1) pathology did not support the diagnosis of ICC, (2) recurrence of ICC, (3) received an anti-tumor therapy before resection, (4) suffering from infectious diseases before resection, (5) suffering from autoimmune diseases or immunodeficiency diseases, (6) patients who died of postoperative complications or reasons other than ICC, (7) R1 or R2 resection, (8) hilar type of ICC, and (9) incomplete clinical data. The ethics committee of Xiangya Hospital of Central South University approved this study.

### Definitions and follow-up

The TNM stage of ICC was determined by the 8th American Joint Committee on Cancer (AJCC) Cancer Staging Manual. NLR was calculated as neutrophil count (10^9^/L)/lymphocyte count (10^9^/L). PLR was calculated as platelet (10^9^/L)/lymphocyte count (10^9^/L), while LMR as lymphocyte count (10^9^/L)/monocyte count (10^9^/L). And the SII was defined as platelet (10^9^/L) × neutrophil/ lymphocyte counts. All patients were received porta hepatis lymphadenectomy during the surgery to determine status of lymph node. Vascular invasion was defined as major vascular invasion.

Patients were followed up every 3 month after surgery. Blood tests including the liver function and tumor biomarkers, and imaging examination was also performed during follow-up. Our primary end points were recurrence-free survival (RFS) and overall survival (OS). RFS was calculated from the first day after hepatectomy to the recurrence of ICC- or ICC-related death, while OS was calculated from the first day after hepatectomy to the ICC-related death.

### Statistical analysis

SPSS 23.0 (SPSS Company, Chicago, IL) for Windows and Prism software (GraphPad Prism Software, La Jolla, CA) were used to analyze data and realize visualization. Independent-sample *t* test or Mann-Whitney *U* test was used to analyze the quantitative data expressed as a mean ± standard deviation (SD). And chi-square or Fisher’s exact test was used as appropriate to analyze the categorical data expressed as frequency (percentage). The cutoff values were calculated by receiver operating characteristic (ROC) curves. Kaplan-Meier curves were used to illustrate RFS and OS, while the log-rank test was used to detect the differences between groups. Meanwhile, the Cox’s proportional hazard regression was used to identify associated factors of RFS and OS. *P* < 0.05 was considered as a statistically significant.

## Results

### Patient and tumor characteristics

128 ICC patients, including 70 males and 58 females, were finally included. The basic patient and tumor characteristics were shown in Table 1. 28.1% of patients presented multiple tumors and 34.4% patients suffered from lymph node metastasis. Proportions of patients with AJCC tumor stages I, II, and III were 26.6%, 14.8%, and 58.6%, respectively. 39.8% patients presented vascular invasion, while 13.3% of patients had undermined the liver function. The averages of alanine aminotransferase (ALT), aspartate aminotransferase (AST), and albumin were 49.58 ± 57.93 U/L, 42.51 ± 31.27 U/L, and 40.33 ± 4.45 g/L. The mean values of CEA, CA19-9, and CA242 were 6.96 ± 15.93 ng/ml, 272.69 ± 318.33 U/ml, and 75.56 ± 104.66 U/ml, respectively. The mean values of neutrophil, lymphocyte, monocyte, and platelet counts were (4.70 ± 1.91)10^9^/L, (1.59 ± 0.49)10^9^/L, (0.66 ± 0.87)10^9^/L, and (232.39 ± 97.68)10^9^/L, respectively. Furthermore, the mean values of calculated NLR, PLR, LMR, and SII were 3.30 ± 2.16, 156.79 ± 77.66, 3.20 ± 2.46, and 793.67 ± 695.14, respectively.

**Table 1:** Clinicopathologic variables of included ICC patients

Variables	Values (*n* = 128)
Age (years)	56.19 ± 9.63
Male	70 (54.7)
Tumor size (cm)	5.83 ± 2.85
Number of tumors	
Single	92 (71.9)
Multiple	36 (28.1)
AJCC tumor stage	
I	34 (26.6)
II	19 (14.8)
III	75 (58.6)
Lymph node metastasis	
No	84 (65.6)
Yes	44 (34.4)
Vascular invasion	
No	77 (60.2)
Yes	51 (39.8)
Tumor differentiation	
Well to moderate	44 (34.4)
Poor to undifferentiated	84 (65.6)
ALT (U/L)	49.58 ± 57.93
AST (U/L)	42.51 ± 31.27
CEA (ng/ml)	6.96 ± 15.93
CA19-9 (U/ml)	272.69 ± 318.33
CA242 (U/ml)	75.56 ± 104.66
Neutrophil (10^9^/L)	4.70 ± 1.91
Lymphocyte (10^9^/L)	1.59 ± 0.49
Monocyte (10^9^/L)	0.66 ± 0.87
PLT (10^9^/L)	232.39 ± 97.68
Albumin (g/L)	40.33 ± 4.45
Child-Pugh score	
A	111 (86.7)
B	17 (13.3)
NLR	3.30 ± 2.16
PLR	156.79 ± 77.66
LMR	3.20 ± 2.46
SII	793.67 ± 695.14

Data are expressed as mean ± standard deviation or *n* (%)

*ICC* intrahepatic cholangiocarcinoma, *AJCC* American Joint Committee on Cancer, *ALT* alanine aminotransferase, *AST* aspartate aminotransferase, *CEA* carcinoembryonic antigen, *PLT* blood platelet, *NLR* neutrophil-to-lymphocyte ratio, *PLR* platelet-to-lymphocyte ratio, *LMR* lymphocyte-to-monocyte ratio, *SII* systemic immune-inflammation index

### Cutoff values of inflammatory biomarkers

**Fig. 1:**
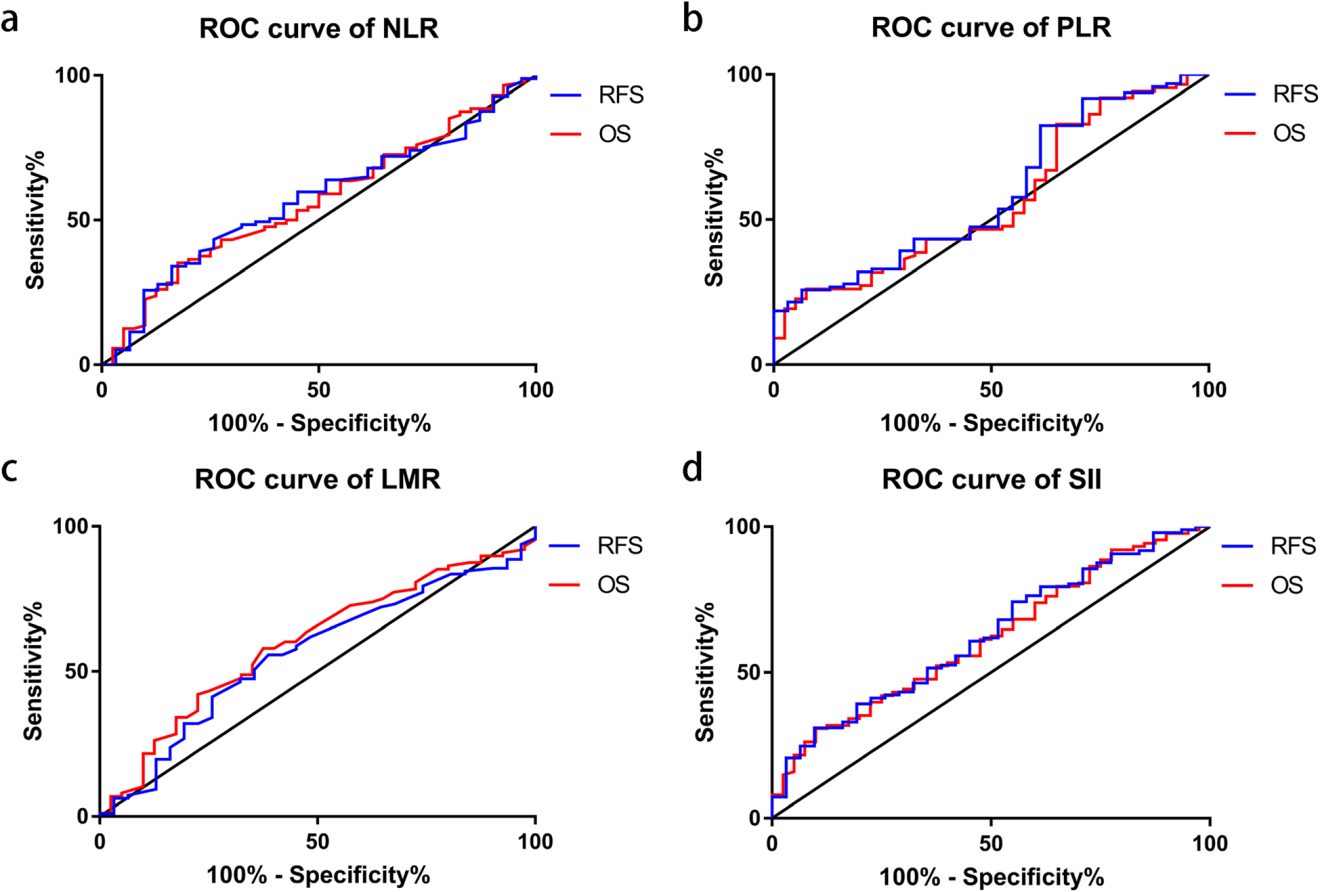
ROC curves of NLR (**a**), PLR (**b**), LMR (**c**), and SII (**d**) on RFS and OS. **a** The AUC for RFS and OS were 0.569 (95%CI = 0.459–0.679, *P* = 0.250) and 0.565 (95%CI = 0.461–0.669, *P* = 0.239). **b** The AUC for RFS and OS were 0.592 (95%CI = 0.477–0.707, *P* = 0.125) and 0.568 (95%CI = 0.460–0.675, *P* = 0.222). **c** The AUC for RFS and OS were 0.561 (95%CI = 0.447–0.674, *P* = 0.310) and 0.597 (95%CI = 0.493–0.701, *P* = 0.079). **d** The AUC for RFS and OS were 0.626 (95%CI = 0.517–0.736, *P* = 0.035) and 0.617 (95%CI = 0.515–0.719, *P* = 0.034). ROC receiver operating characteristic, AUC area under curve, NLR neutrophil-to-lymphocyte ratio, PLR platelet-to-lymphocyte ratio, LMR lymphocyte-to-monocyte ratio, SII systemic immune-inflammation index, RFS recurrence free survival, OS overall survival

ROC curves were performed to determine appropriate cutoff values of NLR, PLR, LMR, and SII, and the results were shown in Fig. 1. However, according to the ROC curves of RFS and OS, the results showed no significance in NLR, PLR, or LMR, while significant results were shown on SII in both RFS (*P* = 0.035) and OS (*P* = 0.034) with areas under ROC curve as 0.63 (95%CI 0.52–0.74) and 0.62 (95%CI 0.51–0.72), respectively. Thus, the subsequent analyses were focused on SII with an ideal cutoff value as 1027 according to ROC curves and Youden index.

### Survival analyses based on SII

**Fig. 2:**
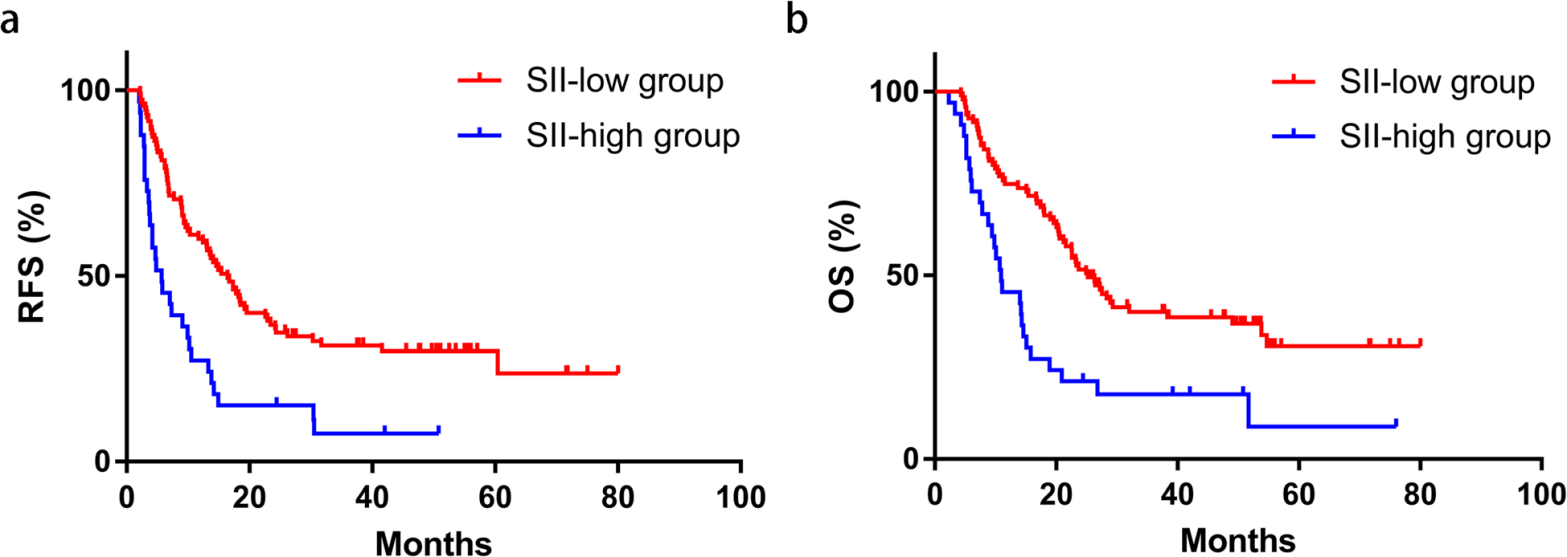
Comparisons between SII-low group and SII-high group using Kaplan-Miere curves on RFS (**a**) and OS (**b**). Better survival data were showed in SII-low group on both RFS (*P* < 0.001) and OS (*P* < 0.001). SII systemic immune-inflammation index, RFS recurrence-free survival, OS overall survival

Survival analyses were performed between SII-low group and SII-high group according to cutoff value of SII, and the results were shown in Fig. 2. The median RFS times in the SII-low group and SII-high group were 16.4 months and 5.7 months, and the median OS times were 25.2 months and 10.9 months, respectively. Statistically significant differences between the two groups were revealed by Kaplan-Meier curves on both RFS (*P* < 0.001) and OS (*P* < 0.001), indicating a potential prognostic value of SII. Only one patient suffered from recurrence after 5 years (60.4 months). Another surgery was performed since the recurrence was intrahepatic, and no new recurrence was found in the last follow-up.

### Univariate and multivariate analyses

For further investigating risk factors affecting RFS and OS of ICC patients, the univariate and multivariate analyses were subsequently performed among available factors, with the results shown in Table 2. The analyses revealed that multiple tumors, higher AJCC tumor stage, poorer tumor differentiation, higher level of CEA and CA19-9, and higher level of SII were independently associated with both poorer RFS time and OS time. However, no significant result was shown on NLR, PLR, or LMR.

**Table 2:** Univariate and multivariate analyses of risk factors with RFS and OS in ICC patients

Variables	RFS		OS	
	HR (95%CI)	*P* value	HR (95%CI)	*P* value
Univariate analyses				
Age (years) (> 60 vs. ≤ 60)	0.670 (0.440, 1.018)	0.061	0.794 (0.514, 1.226)	0.298
Gender (male vs. female)	0.899 (0.603, 1.341)	0.603	0.763 (0.502, 1.161)	0.207
Tumor size (cm)	1.126 (1.053, 1.204)	0.001	1.107 (1.035, 1.186)	0.003
Number of tumors (multiple vs. single)	1.797 (1.168, 2.763)	0.008	1.936 (1.246, 3.008)	0.003
AJCC tumor stage (III vs. I and II)	1.726 (1.135, 2.624)	0.011	1.960 (1.250, 3.074)	0.003
Lymph node metastasis (yes vs. no)	1.881 (1.243, 2.848)	0.003	2.197 (1.430, 3.376)	< 0.001
Vascular invasion (yes vs. no)	1.558 (1.041, 2.332)	0.031	1.478 (0.972, 2.248)	0.068
Tumor differentiation (poor to undifferentiated vs. well to moderate)	2.173 (1.389, 3.401)	0.001	1.971 (1.222, 3.179)	0.005
ALT (U/L)	0.998 (0.994, 1.001)	0.195	0.999 (0.995, 1.003)	0.570
AST (U/L)	0.996 (0.990, 1.003)	0.273	0.999 (0.993, 1.005)	0.815
CEA (ng/ml)	1.020 (1.008, 1.032)	0.001	1.027 (1.015, 1.040)	< 0.001
CA19-9 (U/ml)	1.002 (1.001, 1.002)	< 0.001	1.002 (1.001, 1.003)	< 0.001
CA242 (U/ml)	1.005 (1.003, 1.007)	< 0.001	1.005 (1.003, 1.007)	< 0.001
NLR	1.018 (0.947, 1.096)	0.625	1.033 (0.960, 1.112)	0.387
PLR	1.003 (1.000, 1.005)	0.021	1.003 (1.001, 1.006)	0.011
LMR	1.039 (0.943, 1.146)	0.435	1.019 (0.903, 1.151)	0.757
SII (SII-low group vs. SII-high group)	2.352 (1.519, 3.641)	< 0.001	2.340 (1.484, 3.689)	< 0.001
Albumin (g/L)	0.979 (0.936, 1.023)	0.347	0.969 (0.924, 1.017)	0.203
Child-Pugh score (B vs. A)	0.996 (0.564, 1.757)	0.988	1.286 (0.725, 2.279)	0.389
Multivariate analyses				
Tumor size (cm)	1.030 (0.953, 1.113)	0.456	0.976 (0.899, 1.060)	0.563
Number of tumors (multiple vs. single)	1.849 (1.168, 2.928)	0.009	2.017 (1.274, 3.192)	0.003
AJCC tumor stage (III vs. I and II)	1.599 (1.018, 2.511)	0.042	1.982 (1.207, 3.255)	0.007
Tumor differentiation (poor to undifferentiated vs. well to moderate)	2.355 (1.444, 3.840)	0.001	1.865 (1.093, 3.182)	0.022
CEA (ng/ml)	1.022 (1.008, 1.037)	0.003	1.033 (1.017, 1.049)	< 0.001
CA19-9 (U/ml)	1.001 (1.000, 1.002)	0.034	1.002 (1.001, 1.003)	0.001
CA242 (U/ml)	1.001 (0.998, 1.004)	0.552	0.999 (0.996, 1.003)	0.712
PLR	0.998 (0.995, 1.002)	0.327	0.998 (0.995, 1.002)	0.316
SII (SII-high group vs. SII-low group)	2.368 (1.279, 4.386)	0.006	2.454 (1.278, 4.712)	0.007

*HR* hazard ratio, *CI* confidence interval, *ICC* intrahepatic cholangiocarcinoma, *RFS* recurrence-free survival, *OS* overall survival, *AJCC* American Joint Committee on Cancer, *ALT* alanine aminotransferase, *AST* aspartate aminotransferase, *CEA* carcinoembryonic antigen, *NLR* neutrophil-to-lymphocyte ratio, *PLR* platelet-to-lymphocyte ratio, *LMR* lymphocyte-to-monocyte ratio, *SII* systemic immune-inflammation index

## Discussion

It is widely recognized that the systemic inflammation involves in pathogenesis and progression of cancer by various mechanisms including cell proliferation, tissue infiltration, and angiogenesis [11, 12]. Multiple inflammatory biomarkers could effectively present the extent of inflammatory and immune response with high availability, therefore, were recommend as factors for predicting the prognosis of cancer patients. In the present study, we investigated the prognostic significance of inflammatory biomarkers in curative resected ICC patients. Our results suggested that SII could effectively predict the prognosis of ICC patients after curative hepatectomy, while NLR, PLR, and LMR were not related with outcomes of these patients. This finding could help classifying ICC patients with a highly feasible indicator. Additional treatments, such as radiotherapy and chemotherapy, should be considered to improve the prognosis among patients with poorer prognosis predicted by SII. Furthermore, SII could serve as an indicator for planning therapies, especially immunotherapy, among ICC patients in the future.

Extensive non-specific inflammatory responses were usually led by allogeneic phenotype of cancer cell, followed by increasing of neutrophils and platelets, and deceasing of lymphocytes [13]. Neutrophils could secrete TNF-alpha, VEGF, and interleukin, thus to promote tumor cell proliferation and angiogenesis [14]. Meanwhile, TGF-beta, VEGF, and platelet-derived factors could be secreted by platelets, accelerating differentiation and proliferation of cancer cells, and playing a significant role in adhesion and angiogenesis of tumor tissues. On the other hand, lymphocytes could mediate cytotoxicity and release cytokines, thus presenting antitumor effects as inhibiting growth, proliferation, and metastasis of tumor cell [15]. The decrease of lymphoctyes could lead to lower immune function, progression of tumor, and eventually poor prognosis of patients with tumor. Furthermore, studies showed that activity of lymphocytes could be suppressed by neutrophils [16]. In addition, monocytes in tumor tissues can differentiate into tumor-associated macrophages, which place promoting effects on tumor growth, tumor cell infiltration, and angiogenesis [17]. Thus, the NLR, PLR, LMR, and SII would theoretically be valuable biomarkers for predicting prognosis of cancer, considering all of them could be easily obtained from routine preoperative examinations.

In ICC, this study showed SII as the only independent risk factor on RFS and OS of patients. Two previous studies have also investigated the role of SII in OS among ICC patients [18, 19]. Their results both indicated higher SII was associated with poorer patient survival in ICC, which was consistent with our results. However, one of them also showed that NLR had a better significance as a biomarker on ICC patient. The inconsistent results on NLR might be caused by different cohorts because they did not focus on the patients underwent a curative therapy but the whole ICC cohort.

The present study did contain a few limitations. Firstly, this was a retrospective study with not large sample size. Further prospective, multicenter clinical studies with large cohorts should be performed to validate the values of these inflammatory biomarkers in ICC. Secondly, these inflammatory biomarkers were assessed by single measurements during admission, which might cause uncontrolled bias. Thirdly, some factors which could make an impact on these inflammatory biomarkers, such as smoking and alcoholic, were not fully under control.

## Conclusion

In summary, our study shows SII can effectively predict the prognosis of ICC patient undergone curative hepatectomy, while NLR, PLR, and LMR are not related with clinical outcomes of these patients.

## Supplementary information

**Additional file 1.**

## Data Availability

All data generated or analyzed during this study are included in this published article.

## Abbreviations

ICC: Intrahepatic cholangiocarcinoma
NLR: Neutrophil-to-lymphocyte ratio
PLR: Platelet-to-lymphocyte ratio
LMR: Lymphocyte-to-monocyte ratio
SII: Systemic immune-inflammation index
RFS: Recurrence-free survival
OS: Overall survival
SD: Standard deviation
ROC: Receiver operating characteristic
HBV: Hepatitis B virus
ALT: Alanine aminotransferase
AST: Aspartate aminotransferase
CEA: Carcinoembryonic antigen
